# C-Reactive Protein, International Normalized Ratio, and Fibrinogen in Diagnostic Scale of Complicated Acute Appendicitis

**DOI:** 10.3390/clinpract15020025

**Published:** 2025-01-23

**Authors:** Leticia Lorena Hernández-González, Said José Serrano-Guzmán, Jesús David Guzmán-Ortiz, Hermelo Esteban Pérez-Ceballos, José Luis Cano-Pérez, Víctor Cruz-Hernández, Héctor Ulises Bernardino-Hernández, Lucía Lourdes Martínez-Martínez, Sergio Roberto Aguilar-Ruiz

**Affiliations:** 1 Facultad de Sistemas Biológicos e Innovación Tecnológica, Universidad Autónoma “Benito Juárez” de Oaxaca, Oaxaca 68120, Mexico; leticialorenahg1204@gmail.com (L.L.H.-G.); jcano@uabjo.mx (J.L.C.-P.); 2 División de Cirugía, Hospital General “Dr. Aurelio Valdivieso”, Instituto Mexicano del Seguro Social para el Bienestar, Oaxaca 68050, Mexico; segs941119.fmc@uabjo.mx (S.J.S.-G.); davoguzor@gmail.com (J.D.G.-O.); estebanhdav@gmail.com (H.E.P.-C.); 3 División de Medicina Interna, Hospital General “Dr. Aurelio Valdivieso”, Instituto Mexicano del Seguro Social para el Bienestar, Oaxaca 68050, Mexico; adulvictor@yahoo.com.mx; 4 Facultad de Ciencias Químicas, Universidad Autónoma “Benito Juárez” de Oaxaca, Oaxaca 68120, Mexico; hbernardino@yahoo.com; 5 Facultad de Medicina y Cirugía, Universidad Autónoma “Benito Juárez” de Oaxaca, Oaxaca 68120, Mexico; lmartinez.fmc@uabjo.mx

**Keywords:** complicated acute appendicitis, fibrinogen, C-reactive protein, International Normalized Ratio, scale

## Abstract

**Background/Objectives:** Differentiating complicated acute appendicitis (CA) and uncomplicated acute appendicitis (UC) is essential to guide clinical management. While CA requires urgent surgical management, UC can be treated with antibiotic therapy in selected cases. However, accurate identification of CA remains a clinical challenge. This study aimed to identify factors associated with CA and to develop a diagnostic severity scale. **Methods:** In this retrospective study, we included 132 adult patients (>16 years) with a confirmed postsurgical diagnosis of appendicitis, of whom 52 had CA and 80 had UA. Signs, symptoms, comorbidities, laboratory values, and ultrasonographic findings were evaluated to determine predictive factors and construct a diagnostic scale. **Results:** The factors most significantly associated with CA were elevated plasma concentrations of C-reactive protein (>7.150 mg/dL), fibrinogen (481.5 mg/dL), International Normalized Ratio (INR) (>1.150), and the presence of free fluid periappendicular. The combination of these factors within one scale showed an area under the curve (AUC) of 0.84, with a sensitivity of 78.75% and a specificity of 82.69%. **Conclusions:** Serum C-reactive protein concentration, fibrinogen, and INR can be employed individually or as part of a scale as important indicators in diagnosing CA.

## 1. Introduction

Acute appendicitis is the sudden and severe inflammation of the vermiform appendix and is the most frequent surgical emergency worldwide [1]. Acute appendicitis can be classified as complicated (CA) and uncomplicated (UA). In cases of CA, inflammation progresses, causing venous infarction, gangrene, and perforation, which can lead to abscess formation, peritonitis, and sepsis [2]. In CA, the time to perforation of the appendix is generally between 24 and 48 h after the onset of symptoms, so open or laparoscopic appendectomy is indicated in these cases. On the other hand, UA can be managed with antibiotic therapy, showing promising results and avoiding surgical risk, recovery time, and hospitalization costs [3,4,5,6]. In this context, accurate diagnosis of the severity of acute appendicitis is essential to select the most appropriate treatment [7]. Determining the severity of acute appendicitis remains a clinical challenge [8]. Markers such as C-reactive protein have been proposed and have shown promising results [9,10,11]. Other markers, such as bilirubin, fibrinogen, and procalcitonin, are being explored [12,13].

Imaging tests are fundamental tools for assessing the severity of appendicitis, with computed tomography (CT), magnetic resonance (MR), and ultrasound (US) standing out [14,15]. However, The World Society for Emergency Surgery (WSES) recommends US as the first-line method due to its effectiveness, speed, and low cost [16]. In addition, CT is reserved for cases in which US is inconclusive, as it involves higher radiation exposure than other standard radiological techniques [17]. Similar to CT, MR is effective in detecting complications associated with appendicitis and is also generally not considered a first-line diagnostic tool because of the inaccessibility of this test. In addition, the use of contrast in MR can cause adverse effects, such as nausea, allergic reactions, and renal toxicity [18,19].

Scales that include clinical criteria and hematological markers, such as the Alvarado and Raja Isteri Pengiran Anak Saleha appendicitis (RIPASA) scale, have limitations in differentiating CA from UA [20,21]. Biomarkers are being evaluated to integrate them into current scales. In addition, imaging tests were decided to be included in the scales, such as the AASI (Acute Appendicitis Severity Index) and the Atema score [15,22,23]. However, there is no consensus scale for the detection of CA. This limitation could be related to the variability in clinical presentation. Therefore, the postsurgical report is the gold standard for its determination. For this reason, it is important to analyze the characteristics and risk factors of different population groups. Therefore, this work analyzed demographic variables, signs and symptoms, hematological and biochemical values, coagulation times, and ultrasound findings to elaborate a diagnostic severity scale.

## 2. Materials and Methods

### 2.1. Participants, Variables, and Study Design

This study was retrospective and cross-sectional. It analyzed the clinical records of 132 patients who attended the surgery service of the Hospital Dr. Aurelio Valdivieso, Oaxaca, Mexico, from July 2023 to July 2024. The protocol was authorized by the hospital’s ethics and research committees (HGDAV/CI/0002/2024; approved on 23 July 2024). Inclusion criteria were records of adult patients (>16 years), men and women, with a diagnosis of CA and UA confirmed by a postsurgical histopathological report. CA was defined as gangrenous and/or perforated appendicitis. Data were collected on sex, age, consumption of anti-inflammatory drugs or self-medicated antibiotics before hospital admission, and the presence of comorbidities such as diabetes, hypertension, obesity, and autoimmune diseases. Clinical signs and symptoms include anorexia, pain migration, fever greater than 38 °C, nausea/vomiting, right lower quadrant pain, right iliac fossa rebound, or muscle resistance (Blumberg sign). Laboratory values: leukocyte, neutrophil, monocyte, and platelet counts, mean platelet volume, activated partial thromboplastin time (aPTT), prothrombin time (PT), International Normalized Ratio (INR), C-reactive protein, and fibrinogen. Ultrasound findings: appendicolith, periappendicular plastontium, abscess, and free fluid in periappendicular cavity. Finally, the Neutrophil/Lymphocyte Ratio (NLR), Platelet/Lymphocyte Ratio (PLR), and Monocyte/Lymphocyte Ratio (MLR). The scores obtained from the Alvarado and RIPASA scales were also recorded. Data were discarded from incomplete records, as well as patients with the following conditions: pregnant women, immunodeficiencies, viral or bacterial infections, cancer or receiving chemotherapy, bleeding, liver disease, and those who recently received transfusions, and other inflammatory conditions that may affect hematological values.

### 2.2. Statistical Analysis

The data were entered into an electronic spreadsheet for analysis. Descriptive analysis was applied to the data, using means plus standard deviations or percentages according to the variables’ nature. The normality of the data was determined with the Kolmogorov–Smirnov test. We used independent Student’s *t* o U de Mann-Whitney to compare means and distribution of ranks and chi-square for proportions. All analyses were performed with GraphPad Prism version 8.0.2 (Boston, MA, USA). Binomial logistic regression (LR) analysis was performed with IBM SPSS statistics 27.0 (Armonk, NY, USA), considering the significantly different variables between CA and UA from the previous analysis. Due to the variability in the concentration of C-reactive protein, we categorized its concentration into 1 = concentration greater than 9 mg/dL and 0 = concentration less than 9 mg/dL for analysis in the LR (the value of 9 mg/dL is used in the laboratory reports of our hospital). The binomial dependent variable was 0 = absence and 1 = presence, with the postoperative diagnosis of CA as presence. The predictor variables obtained from the LR were used to generate a model from which we developed the diagnostic severity scale.

### 2.3. Construction of the Score

In order to facilitate the application of our results in clinical practice, continuous variables were converted to categorical variables using ROC curve analysis (Receiver Operating Characteristic), which allowed us to obtain the best points to differentiate patients with CA from uncomplicated patients; we obtained the cutoff point using Youden’s index with 0 assigned to cases below the cutoff point and 1 assigned to those equal to or greater than the cutoff point. We assigned the scores using the natural logarithm (ln) of the odds ratio, k = 2, and rounded to the nearest whole number for ease of application. We then evaluated the performance of our scale and compared it with the scores of the Alvarado and RIPASA scales [24,25], and the NLR, PLR, and MLR indices using ROC curve analysis. In all tests performed, a value of *p* < 0.05 was considered to indicate a statistically significant difference. In addition, 95% confidence intervals (CI) were calculated using the same level of significance (*p* < 0.05) to ensure the accuracy of the results.

## 3. Results

### 3.1. Comparison of Preoperative Factors Between Complicated and Uncomplicated Acute Appendicitis

Initially, we classified the records of patients diagnosed with appendicitis as follows. The initial sample obtained was 311 records of patients diagnosed with acute appendicitis, of which 179 were excluded for not meeting the inclusion criteria (materials and methods). Since analyzing C-reactive protein concentration implies an increase in the cost of the service, this parameter is not routinely measured. For this reason, the high exclusion rate was mainly due to the lack of recording of this parameter. Of the 132 records selected, we found that 52 patients were diagnosed with CA and 80 with UA. Of these, only one patient underwent laparoscopic surgery, and all the others underwent open surgery. Of the 40 variables measured and analyzed, 14 significantly differed between both groups (Table 1). The variables that showed a significant difference (*p*-value < 0.05) between CA and UA correspond to gender, fever greater than 38 °C, leukocytes, neutrophils, fibrinogen, C-reactive protein, prothrombin time (PT), INR, abscess, free fluid in perpendicular cavity, NLR, PLR, and MLR, and RIPASA point scales.

### 3.2. Factors Associated with Complicated Acute Appendicitis

LR was used to evaluate the main independent predictive variables or risk factors for CA. The variables evaluated were those that showed significant differences between CA and UA in the previous analysis, excluding those with insufficient sample numbers, and between the concentration of leukocytes and neutrophils; only the latter was considered to avoid redundancy. The results found show that fibrinogen (Odds Ratio, O.R. 8.277; *p* = 0.004), C-reactive protein (O.R. 7.533; *p* = 0.006), INR (O.R. 4.651; *p* = 0.031), and free fluid (O.R. 4.612; *p* = 0.032), were the most important predictor variables (Table 2).

### 3.3. Plasma Concentrations of C-Reactive Protein, Fibrinogen, and INR Are Individual Factors Associated with CA

**Figure 1:**
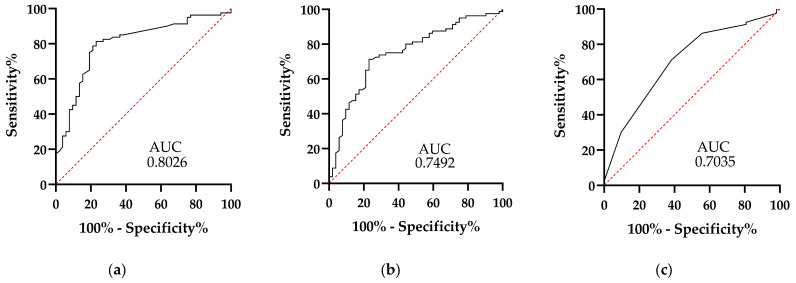
ROC curves of plasma concentrations of C-reactive protein (**a**), fibrinogen (**b**), and INR (**c**).

It was found that serum concentrations of C-reactive protein and fibrinogen, as well as INR and free fluid, were the variables shown to have the strongest association with CA. Our next objective was to determine these variables’ discriminative power, specificity, and sensitivity from the ROC curve analysis using a 95% CI (confidence interval). It is worth mentioning that this procedure was not applied to the free fluid data because this is a nominal variable. The results obtained for serum C-reactive protein concentrations are an area under the curve (AUC) of 0.8026 with a range of 0.7246 to 0.8807, a cutoff point of 7.150 mg/dL, a sensitivity of 78.75%, and a 78.85% specificity (Figure 1a). The results for serum fibrinogen concentrations are an AUC of 0.7492 with a range of 0.6635 to 0.8348, a cutoff point of 481.5 mg/dL, with 72.5% sensitivity and 73.08% specificity (Figure 1b). Finally, INR has an AUC of 0.7035, a range of 0.6124 to 0.7945, and a cutoff point of 1.150, with 71.25% sensitivity and 61.54% specificity (Figure 1c). In conclusion, our results indicate that plasma concentrations of C-reactive protein, fibrinogen, and INR are individual factors that can be used to diagnose CA.

### 3.4. Diagnostic Scale for Complicated Acute Appendicitis

**Figure 2:**
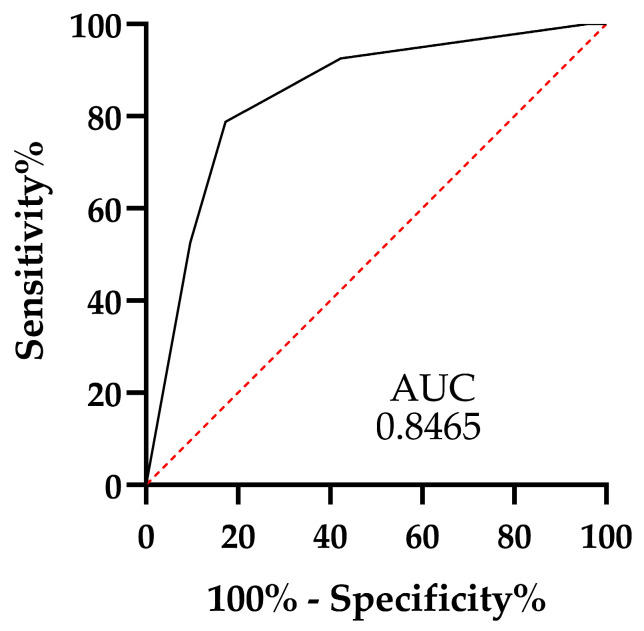
The ROC curve for the diagnosis of CA was created from plasma concentrations of C-reactive protein and fibrinogen, INR, and free liquid.

After determining that serum concentrations of C-reactive protein, fibrinogen, and INR are the main predictors of CA in our study, we decided to include them in a model that would allow us to differentiate CA from UA adequately. We also added free fluid to our model in the periappendicular cavity (due to its relationship with CA in the LR). In addition, variables were categorized into 0 = below cutoff and 1 = above cutoff obtained from ROC curve analysis; the cutoff for C-reactive protein, fibrinogen, and INR was the point where it reached the highest sensitivity and specificity and was determined using Youden’s index for each of these parameters. Our model was analyzed using LR. The results show that all the variables evaluated are explanatory of CA. We assigned the score using the odds ratio and rounding to the nearest integer to facilitate its application (Table 3). The area under the curve of the proposed scale was 0.8465, with a range of 0.7748 to 0.9182 and a *p* = 95%. The optimal cutoff point was 3.0, with a sensitivity of 78.75% and a specificity of 82.69% (Figure 2).

### 3.5. Performance of the Scale Generated

Finally, we decided to compare the performance of our scale with that of other currently used scales and ratios. The results of the AUC were as follows: NLR (AUC: 0.6647), PLR (AUC: 0.6512), MLR (AUC: 0.6242), Alvarado (AUC: 0.5958), and RIPASA (AUC: 0.6457) scales (Table 4). In conclusion, our scale has a higher AUC, sensitivity, and specificity than other scales based primarily on the use of inflammatory factors and signs and symptoms, such as the Alvarado and RIPASA scales.

## 4. Discussion

Acute appendicitis is the most common surgical emergency worldwide [1]. Being able to discern between CA and UA is of great importance because while CA requires immediate surgical attention, UA can be treated with antibiotic therapy [6]. Imaging tools, such as computed tomography (CT), magnetic resonance (MR), and ultrasound (US), are the most common techniques to identify the severity of appendicitis [8,26]. On the other hand, NLR, PLR, and MLR ratios, as well as inflammatory markers, C-reactive protein, bilirubin, and fibrinogen, have also proven helpful in the differential diagnosis of CA and UA [9,10,11,12,13]. Nevertheless, the individual use of imaging techniques or inflammatory markers does not allow a conclusive diagnosis [27,28]. The World Society for Emergency Surgery (WSES) recommends combining clinical and imaging values to diagnose CA [16]. The implementation of scales that integrate markers such as C-reactive protein and imaging is helpful [9,29]. There is no universally accepted scale; therefore, further research is needed to identify new useful variables or validate existing scales. This work analyzes different factors and proposes a diagnostic scale to assess the severity of acute appendicitis.

Our results indicate that several factors are associated with complicated acute appendicitis. These include increased serum fibrinogen and C-reactive protein concentration, international normalized ratio, and US findings such as free fluid. The construction of our diagnostic scale based on these parameters could help identify this complication, as demonstrated by the overall efficiency obtained (AUC of 0.84), with a sensitivity of 78.75% and a specificity of 82.69%. Overall, our scale achieved levels of sensitivity and specificity that are among the highest reported in scales without and with TC support [30,31]. The high sensitivity and specificity achieved with our scale play a key role in clinical decision-making. For example, high sensitivity is crucial for initiating emergency protocols, while high specificity in diagnostic testing can help prevent unnecessary surgical interventions.

The increased diagnostic power of our scale is due to the incorporation of C-reactive protein, fibrinogen, and INR. The results of this study prove that the serum concentration of C-reactive protein alone has a high power to discern between CA and UA. C-reactive protein is a pentraxin with key functions in recognizing and opsonizing pathogens and activating the complement system [32]. The liver mainly produces it in response to acute and chronic inflammatory states. Upon activation, C-reactive protein is cleaved into various isoforms, although the pentameric form is the one that has historically been used as a marker in the diagnosis and monitoring of inflammatory diseases [33,34]. However, this marker has limitations in the early stages of appendicitis, as its values may overlap with those of other inflammatory conditions. Nevertheless, this marker is useful in differentiating between complicated and uncomplicated appendicitis [35,36].

The findings of our study highlight that increased fibrinogen concentration is associated with complicated appendicitis. These results agree with those reported by Wu et al. [13] in a meta-analysis that included seven studies, which show that an increase in this molecule is associated with a higher incidence of complicated appendicitis. Fibrinogen, or coagulation factor I, is a plasma glycoprotein. Like C-reactive protein, it is synthesized in the liver. Fibrinogen plays a crucial role in activating the extrinsic coagulation pathway by binding to its primary receptor, platelet glycoprotein IIb/IIIa, which allows clot formation. In addition to its role in hemostasis, fibrinogen can be recognized by integrins such as CD11c/CD18 and CD11b/CD18 (complement receptor 3), which are present in activated neutrophils. This recognition promotes the release of proinflammatory cytokines, such as tumor necrosis factor-alpha (TNF-α) and interleukin 1β (IL-1β) [37,38]. In acute inflammatory states, the plasma concentration of fibrinogen tends to increase significantly, positioning it as a key mediator in acute inflammation [39]. Although the role of fibrinogen in detecting complicated appendicitis is recognized, studies evaluating this marker are scarce [13]. Our study incorporated fibrinogen and INR into a screening scale for the first time, obtaining promising results. WSES highlights that biochemical markers represent a reliable and promising diagnostic tool for identifying complicated acute appendicitis in adults. However, they point out that more high-quality tests are still required [16].

Our analyses indicate that increased del INR and prothrombin time (PT) constitute a risk factor for complications of appendicitis and represent a valuable tool for the detection of complicated appendicitis. This finding is consistent with the study of Morandi et al. [40], who analyzed PT concentration in a pediatric population. Also, Kim et al. [41] reported that INR is a predictive marker of complications in acute appendicitis. However, to date, no additional evidence exploring the relationship between PT or INR and complications of appendicitis is available.

Regarding imaging techniques, in this study, we identified that free fluid peri appendiceal is found in the US that can help identify AC. US is a fast, safe, and reliable technique; when the appendix is wholly visualized, ultrasound can be as sensitive, specific, and accurate as CT. However, its efficacy depends largely on the operator’s experience [42]. Although CT tomography generally has greater diagnostic power, it is considered a second-line tool for the diagnosis of appendicitis and even third line in children [43] due to the exposure to higher radiation doses compared to other typical radiological techniques, despite the implementation of ultra-low-dose strategies [44]. The WSES recommends performing US first in young patients and resorting to CT only in case of negative or inconclusive ultrasound findings [16]. In our study, the mean age for acute appendicitis was 33 years, considered young adults, so it is important to consider its use in similar populations. MRI is an imaging test option in cases where radiation exposure is especially harmful, such as in children and pregnant women [8]. However, the long scanning time and its limited accessibility are far from the first line of emergency diagnosis, indicated after an inconclusive ultrasound scan [16].

Some scales have been developed, combining clinical and imaging factors, such as the Atema score or appendicitis severity scoring system (SAS), which combines computed tomography (CT) images, C-reactive protein, and leukocyte count, and the Appendicitis Severity Index (APSI), which combines clinical features with CT features [45]. However, this tool showed good sensitivity and specificity. Several validation studies have questioned its diagnostic utility, recommending a reevaluation of its applicability [46]. On the other hand, recent modifications to SAS 2.0 are being explored to improve diagnostic accuracy and its clinical use feasibility [23]. However, these scales base their diagnostic power on CT, which has drawbacks. The scale proposed in this work is comparable with others that integrate clinical data and US images, such as the one developed by Atema et al. called Atema–US [30], which achieved an AUC of 0.82. However, its clinical implementation may be limited due to its complexity since it uses a 19-point scale. Biochemical markers are now recognized as valuable tools for determining the complications of appendicitis. Unlike the Atema–US scale, which is limited to using C-reactive protein as a biochemical marker, we also incorporate fibrinogen as an additional biochemical parameter in our scale [16]. One of the main characteristics of the proposed scale is its low cost and benefit since its main variables, such as ultrasound and prothrombin time (necessary to calculate the INR), are routine preoperative studies for the diagnosis of appendicitis, performed in most government hospitals, which will not affect the patient’s economy [47,48]. Although plasma fibrinogen concentration is not a mandatory preoperative parameter, it is usually part of coagulation profiles [40,49]. Finally, C-reactive protein is not mandatory for diagnosing appendicitis, but it is relevant to determining the severity of different inflammatory conditions [41], being an accessible biomarker.

On the other hand, of the 132 operated patients, 11.36% returned due to postoperative complications, 13.43% of patients with complicated acute appendicitis returned due to complications, and 10% of patients who had uncomplicated acute appendicitis remitted; there was no statistical difference between AC and UA. In this parameter, the predominant complications were surgical-site infection, intra-abdominal abscess, fluid collection, and intestinal obstruction. Preoperative hematological markers were evaluated only to assess the risk of intraoperative complications, not for the selection of surgical intervention. An important limitation is that it is a retrospective study, and we could not control the variables collected, such as the time of symptom onset. In this sense, Van Dijik et al. [50] report in a large number of patients that postponing surgery in patients with UA for 24 h does not culminate in CA. Another study shows that in patients with CA, an early appendectomy should be performed within 8 h, suggesting that even both appendicitis may be entities with different etiology and clinical development [51].

Although INR and fibrinogen were significant diagnostic markers, other coagulation factors, such as factors VII, X, and V, which could influence INR values, were not measured. One of the main negative factors of this work is the low number of patients. However, other methodologically related works using a similar number of samples obtained statistically sustained results [52,53]. Another area for improvement of this work is that we cannot generalize the results. We emphasize the need to validate this scale with data from other hospitals to take it to clinical practice [54]. Also, we recommend considering the study of other biochemical markers for the detection of complications of appendicitis for incorporation into diagnostic severity scales.

## 5. Conclusions

This work shows the individual diagnostic power of serum C-reactive protein concentration, fibrinogen, and INR in detecting the severity of appendicitis. In this study, we incorporate these three variables into a diagnostic scale for the first time. Both individually and in combination, these factors show promise for the differential diagnosis of complicated and uncomplicated acute appendicitis.

## Figures and Tables

**Table 1:** Comparison of preoperative factors between acute complicated appendicitis and uncomplicated appendicitis groups.

Variable	CA (n = 52)	UC (n = 80)	*p*-Value
Men % (n) Women % (n)	61.54% (32) 38.46% (20)	41.25% (33) 58.75% (47)	0.0227 (*)
Age (years)	31.67 ± 17.48	34.85 ± 16.61	0.1276
Self-medication with antibiotics	25.00% (13)	14.44% (13)	0.1171
Self-medication with steroidal anti-inflammatory drugs	61.54% (32)	55.00% (44)	0.4577
	Comorbidities		
Diabetes % (n)	5.77% (3)	5% (4)	0.8472
Obesity % (n)	19.23% (10)	12.50% (10)	1.111
Hypertension % (n)	5.77% (3)	2.50% (2)	0.9242
Autoimmune disease % (n)	0	0	-
	Signs and symptoms		
Anorexia	76.92% (40)	67.50% (54)	0.2427
Migration of pain	80.77% (42)	77.50% (62)	0.6535
Fever greater than 38 °C	61.54% (32)	41.25% (33)	0.0227 (*)
Nausea/Vomiting	84.62% (44)	81.25% (65)	0.6184
Right lower quadrant pain	98.08% (51)	100% (80)	0.2131
Right iliac fossa rebound (Blumberg’s sign)	96.15% (50)	86.25% (69)	0.0621
	Laboratory values		
Leukocytes (10^3^/µL)	16.00 ± 6.119	13.88 ± 5.466	0.0393 (*)
Neutrophils (10^3^/µL)	13.55 ± 6.183	11 ± 5.466	0.0142 (*)
Lymphocytes (10^3^/µL)	1.432 ± 1.244	1.693 ± 0.7639	0.1377
Monocytes (10^3/^µL)	0.9737 ± 0.5049	0.9031 ± 0.4235	0.3880
Platelets (10^3^/µL)	273.6 ± 85.07	262 ± 69.66	0.3943
MPV	10.08 ± 1.058	10.15 ± 1.001	0.6997
aPTT(s)	29.14 ± 3.356	28.95 ± 3.451	0.9325
PT(s)	15.99 ± 2.390	14.67 ± 1.551	0.0002 (***)
INR	1.233 ± 0.1978	1.122 ± 0.1321	0.0001 (****)
Fibrinogen (mg/dL)	577.1 ± 165.7	438.3 ± 158.4	<0.0001 (****)
C-reactive protein (mg/dL)	11.69 ± 8.816	6.872 ± 14.32	0.0318 (*)
Total protein (g/dL) ^†^	7.410 ± 2.677	7.214 ± 1.326	0.6836
Albumin (g/dL) ^†^	4.090 ± 0.6973	4.228 ± 0.5568	0.4215
Globulin (g/dL) ^†^	3.275 ± 0.4153	3.192 ± 0.3434	0.4233
Total Bilirubin (g/dL) ^†^	1.185 ± 0.5724	0.9800 ± 0.6067	0.2242
Direct bilirubin (g/dL) ^†^	0.01500 ± 0.06708	0.000 ± 0.000	0.3571
Indirect bilirubin (g/dL) ^†^	1.170 ± 0.5507	0.9778 ± 0.5981	0.2414
	Ultrasound findings		
Appendicolith	3.85% (2)	1.25% (1)	0.3281
Periappendicular Plastontium	17.31% (9)	11.25% (9)	0.3217
Abscess	9.62% (5)	1.25% (1)	0.0242 (*)
Free fluid in periappendicular cavity	13.46% (7)	2.50% (2)	0.0146 (*)
	Scales and ratios		
Alvarado scale points	7.904 ± 1.376	7.375 ± 1.594	0.0513
RIPASA scale point	7.394 ± 1.439	6.669 ± 1.522	0.0043 (**)
NLR	13.26 ± 10.56	8.800 ± 7.338	0.0013 (**)
PLR	255.92 ± 174.4	188.3 ± 97.95	0.0032 (**)
MLR	0.9095 ± 0.7878	0.6668 ± 0.5074	0.0158 (*)
	0.9095 ± 0.7878	0.6668 ± 0.5074	0.0158 (*)

MPV: Mean Platelet Volume, aPTT: activated Partial Thromboplastin Clotting Time, PT: Prothrombin Time, INR: International Normalized Ratio, NLR: Neutrophil/Lymphocyte Ratio, PLR: Platelet/Lymphocyte Ratio, MLR: Monocyte/Lymphocyte Ratio. Values represent percentages and means ± standard deviations. Statistical analysis was performed by Student’s *t*, U de Mann-Whitney o chi-square, with significance indicated as follows: * *p* < 0.05, ** *p* < 0.01, *** *p* < 0.001, **** *p* < 0.0001. ^†^ n < 132.

**Table 2:** Factors associated with complicated acute appendicitis.

Variable	Odds Ratio	*p* Value
Biological sex (male)	0.904	0.342
Fever greater than 38 °C	0.571	0.450
Free fluid	4.612	0.032 (*)
Abscess	3.651	0.056
Neutrophils count	0.599	0.439
Fibrinogen	8.277	0.004 (**)
INR	4.651	0.031 (*)
C-reactive protein	7.533	0.006 (**)
NLR	0.339	0.560
PLR	1.055	0.304
MLR	2.903	0.088
RIPASA score	3.644	0.056

INR: International Normalized Ratio, NLR: Neutrophil/Lymphocyte Ratio, PLR: Platelet/Lymphocesyte Ratio, MLR: Monocyte/Lymphocyte Ratio, RIPASA: Raja Isteri Pengiran Anak Saleha appendicitis. Statistical significance * *p* < 0.05, ** *p* < 0.01.

**Table 3:** Scale for diagnosis of complicated acute appendicitis.

Variables	Clinical Features	Odds Ratio	Significance	Score
	Fibrinogen > 481.5 mg/dL	4.692	0.030 (*)	2
	INR > 1.15	4.479	0.034 (*)	2
	C-reactive protein > 7.15 mg/dL	4.535	0.033 (*)	2
	Free fluid	3.861	0.049 (*)	2
Total score				8

* Statistical significance (*p* < 0.05).

**Table 4:** AUC, Sensitivities, Specificities for cutoff values of NLR, MLR, PLR, Alvarado, and RIPASA scale.

	AUC	C.I. 95% L.L.	C.I. 95% U.L.	Cutoff Point	Sensitivity	Specificity
NLR	0.6647	0.5718	0.7575	9.342	61.25	59.62
MLR	0.6242	0.5259	0.7224	0.6141	61.25	57.69
PLR	0.6512	0.5562	0.7462	203.0	62.50	63.46
Alvarado scale	0.5958	0.4982	0.6934	7.500	51.25	63.46
RIPASA scale	0.6457	0.5498	0.7416	7.250	65	63.46
scale proposed	0.8465	0.7748	0.9182	3.0	78.75	82.69

## Data Availability

The data presented in this study are available on request from the corresponding author.
